# Sirtuins—The New Important Players in Women’s Gynecological Health

**DOI:** 10.3390/antiox10010084

**Published:** 2021-01-10

**Authors:** Ewa Maria Kratz, Izabela Kokot, Violetta Dymicka-Piekarska, Agnieszka Piwowar

**Affiliations:** 1 Department of Laboratory Diagnostics, Division of Laboratory Diagnostics, Faculty of Pharmacy, Wroclaw Medical University, Borowska Street 211A, 50-556 Wroclaw, Poland; ewa.kratz@umed.wroc.pl; 2 Department of Clinical Laboratory Diagnostics, Medical University of Bialystok, Waszyngtona Street 15A, 15-269 Bialystok, Poland; violetta.dymicka-piekarska@umb.edu.pl; 3 Department of Toxicology, Faculty of Pharmacy, Wroclaw Medical University, Borowska Street 211, 50-556 Wroclaw, Poland; agnieszka.piwowar@umed.wroc.pl

**Keywords:** sirtuins, women’s gynecological health, markers of gynecological diseases, oxidative stress

## Abstract

The participation of sirtuins in the regulation of oxidative stress and inflammation lies at the basis of their possible modes of action and is related to their expression in various cell structures; their location in the mitochondria and blood plasma has been indicated as of primary importance. Despite many existing studies, research on sirtuins continues to present an opportunity to discover new functions and dependencies, especially when it comes to women’s gynecological health. Sirtuins have a significant role in both the formation and the course of many gynecological diseases. Their role is particularly important and well documented in the course of the development of cancer within the female reproductive organs; however, disturbances observed in the ovary and oocyte as well as in follicular fluid are also widely investigated. Additionally, sirtuins take part in some gynecological disturbances as regulative factors in pathways associated with insulin resistance, glucose and lipids metabolism disorders. In this review, we would like to summarize the existing knowledge about sirtuins in the manner outlined above.

## 1. Introduction

Women’s gynecological diseases are related to disorders in the female genital or reproductive organs or mammary glands. This includes such disease entities as: vulvitis, vaginal infections, adnexitis, cervical erosion, uterine fibroids, ovarian cyst, and sexually transmitted diseases as well as menstrual disorders, polycystic ovary syndrome (PCOS), endometriosis, and fertility disorders. Additionally, tumor diseases, such as cervical cancer, ovarian cancer, and breast cancer, can be also included in this group. Body weight as well as the impact of some exogenous factors (e.g., xsenoestrogens) can also be the cause of female infertility [1,2,3].

Gynecological morbidities include conditions of the reproductive tract which are not associated with a particular pregnancy, such as reproductive tract infections, cervical cell changes, prolapse, infertility, and related morbidities, including urinary tract infections, but may be related to sexual behavior [4,5]. A systematic review and meta-analysis conducted by Dheresa et al. [6] among women of reproductive age revealed an overall pooled random effect prevalence of gynecological morbidity at 22%. These results indicate that the pooled prevalence of overall gynecological morbidity is high, and the authors have concluded that the effects of high gynecological morbidities cause lower fertility in women of reproductive age around the world, especially in developing nations [6]. Similarly, Rasool et al. [7] documented that, in a population of female inhabitants of the Srinagar district in the Kashmir valley (India), 20.28% reported one or more gynecological symptoms. This indicates the importance of the problem, which has a great impact on women’s health [7]. Due to their complexity, the pathogenesis of gynecological diseases is also multifaceted, but redox status and energy perturbations related to aging and metabolic dysfunctions have recently been indicated [8,9,10]. Some authors believe that, in the future, the combination of different metabolites and other blood-based molecular sets of parameters, such as DNA methylation, microRNA, and cell-free DNA mutation markers, will be an attractive option in diagnostics and monitoring of some female-specific diseases, especially breast cancer [11]. In this light, it seems interesting to examine the role of a specific class of enzymes, known as sirtuins (SIRTs). Sirtuins are able to modify DNA or other proteins by means of their deacetylation. Some authors indicate the biological role of SIRTs in endometriosis, ovarian carcinoma (OvCa), or female and male infertility [12,13,14], which makes them promising diagnostic and therapeutic markers.

## 2. Materials and Methods

A review of the literature was conducted in order to identify the most relevant studies reported, mainly in English. Publications were searched for in the PubMed MEDLINE electronic database. We focused primarily on the activity of sirtuins in the context of women’s gynecological health, including investigations on female animal models and cell lines. The keywords used to search the necessary information were as follows: “sirtuins” and “deacetylases” in various combinations with “gynecological diseases”, “endometriosis,” “polycystic ovary syndrome”, “cancer”, “female infertility”, “oocytes”, “oxidative stress”, “inflammation”, “obesity”, and “steroid hormones”. Moreover, references in each article were searched to identify potentially missed studies.

## 3. Sirtuins

**Figure 1:**
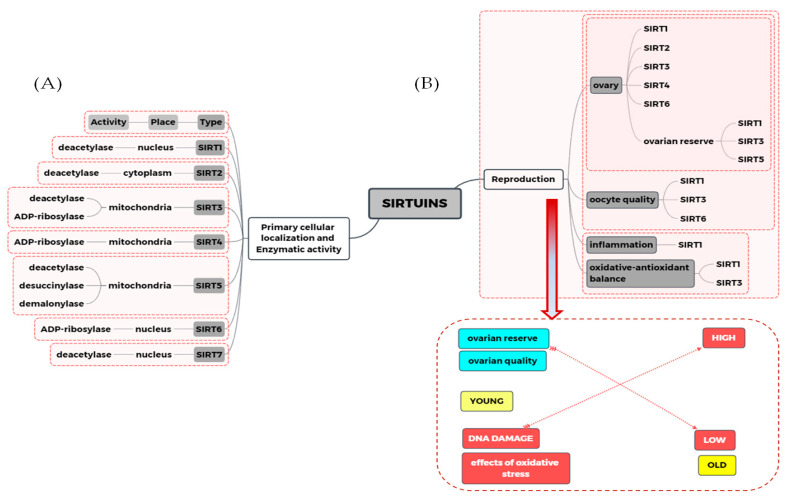
The relationship between sirtuins action and mechanisms involved in the women reproduction process. (**A**) The enzymatic activity and primary cellular location of each sirtuin. (**B**) Summary of the importance of sirtuins in selected mechanisms related to female reproductive health, including ovarian quality, ovarian reserve, and oocyte quality. It was also shown the relationship between oxidative stress, inflammation, and reproductive capacity related to the aging of the ovaries.

**Figure 2:**
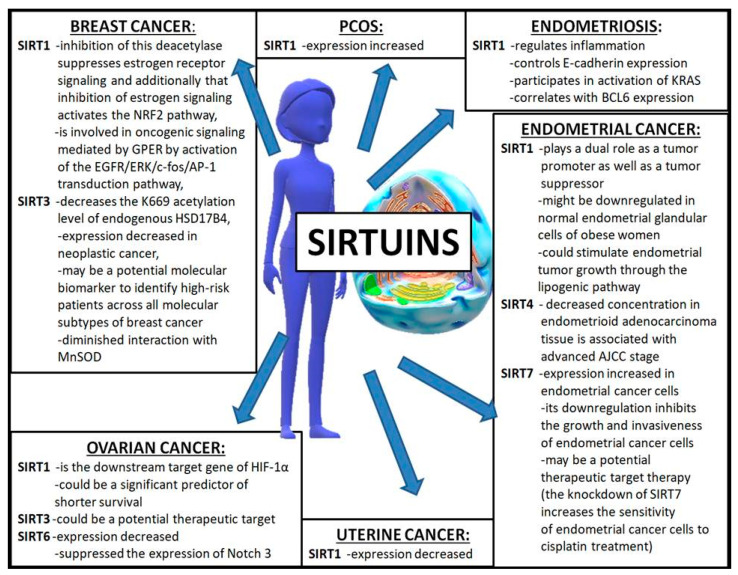
The influence of sirtuins on the development of gynecological diseases. AJCC—American Joint Committee on Cancer; AP-1—activating protein-1; BCL6—B-cell lymphoma 6 protein; c-fos—a proto-oncogene; EGFR—epidermal growth factor receptor; ERK—extracellular signal-regulated kinases; GPER—G-protein ER; HIF-1α—hypoxia-induced factor 1 alpha; HSD17B4—17β-hydroxysteroid dehydrogenase type 4; KRAS—Kirsten rat sarcoma virus; K669—lysine 669; MnSOD—manganese superoxide dismutase; Notch 3—notch receptor 3; NRF2—nuclear factor erythroid 2-related Figure 2. NF-E2-related factor 2.

Sirtuins are class III deacetylases activated by NAD^+^ with different beneficial effects brought by the modification of the structure and function mainly of the DNA histone as well as many other proteins or formations of complexes with proteins. In humans, the family of sirtuins is comprised of seven members (SIRT1—SIRT7). Most sirtuins (1, 2, 3, 5, and 6) exhibit NAD^+^-dependent deacetylase activity. Sirtuins 4 and 6 act as mono-ADP-ribosyl transferase, whereas SIRT3 exhibits both of these activities. Additionally, SIRT5 has the widest range of activity—apart from deacetylation, it can also cause demalonylation and desuccinylation. Some sirtuins are originally present in the nucleus (SIRT1, SIRT6, and SIRT7), in mitochondria (SIRT3, SIRT4, SIRT5) or in cytosol (SIRT2), but they can become relocated during cell differentiation. For example, SIRT6 is present in nucleoli during G1 but not in the S phase of the cell cycle, and slows down mitosis when overexpressed [15]. Information on the cellular localization of sirtuins and their enzymatic activity is gathered and graphically presented in Figure 1. There are many existing studies concerning its structure, function, action, substrates, and modulatory agents [16,17,18,19,20,21], therefore this will not be described in detail in this study. Sirtuins exhibit a multiplicity of various activities in most tissues in the mammal’s organism. The best known and described role of SIRT1, which is present in the liver, skeletal muscle, adipose tissue, pancreas, and brain, is its participation in the regulation of glucose and lipid metabolism, insulin secretion, energy intake, oxidative balance regulation, and immune system action. It has a known role in the pathogenesis of type 2 diabetes, obesity, cardiovascular disease, inflammation and arthritis, osteoporosis, neurodegenerative diseases, and cancer as well as aging and fragility [22,23,24]. Additionally, the role of sirtuins in the reproductive function of men and women as well as in gynecological morbidity is reported [6,15,25,26]. It has been documented that sirtuins are involved in the control of ovarian functions on various regulatory levels (proliferation, apoptosis, secretory activity of ovarian cells, their response to upstream hormonal regulators, ovarian folliculo- and oogenesis, and fecundity). External and endocrine factors can affect female reproduction by targeting the ovarian microenvironment during an entire reproductive lifespan, also via sirtuins [1,27]. Their participation in the regulation of oxidative stress (OS) and inflammation is emphasized as a possible method of action of sirtuins, connected with their expression in various cell structures, but it is mainly their mitochondrial and blood plasma localization that has been indicated as of primary importance, especially in terms of disturbances observed in ovary and oocyte, follicular fluid (FF), and cancer development. Additionally, their regulative role in pathways associated with insulin resistance, glucose, and lipid metabolism disorders, which are connected with polycystic ovary syndrome occurrence, is also documented [1,13,25,28,29,30,31]. The influence of sirtuins on the development of gynecological diseases is shown in Figure 1 and Figure 2, and all sirtuins that influence women’s gynecological health have been gathered in Table 1.

## 4. Sirtuins and Female Fertility

Problems with female fertility are complex and connected to a broad spectrum of disturbances of basic ovarian functions (proliferation, apoptosis, secretory activity of ovarian cells, their response to upstream hormonal regulators, ovarian folliculo- and oogenesis). Moreover, with advancing female age, there has been a gradual decline in the size of the ovarian follicle pool, oocyte yield, and oocyte quality. This process has been termed ovarian ageing [34].

Recently, Bódis et al. [34] simultaneously examined serum and follicular fluid levels of SIRT1 and SIRT6 in healthy women with normal body weight and several years of fertility problems, who had undergone in vitro fertilization, whether it resulted in a successful pregnancy or not. Moreover, the resveratrol (RSV) level, being a known activator of sirtuins, was examined. It was indicated that resveratrol protects against age-associated infertility and improves oocyte maturation and subsequent development in various mammals [57,58,59,60]. The authors reported that ovarian hyperstimulation resulted in significantly higher serum SIRT1 levels in pregnant women compared with non-pregnant women. However, SIRT6 levels remained unchanged after ovarian hyperstimulation but were significantly lower in pregnant women compared with non-pregnant women (before and after hyperstimulation). Both sirtuins were detected in follicular fluid, but their concentrations were lower than in serum and seemed to be independent for both these body fluids. The authors also showed that follicular fluid levels of SIRT6 were positively related to mature oocytes, whereas serum levels of both sirtuins were related to clinical pregnancy. The level of resveratrol was markedly lower in follicular fluid than in serum, and its level was higher in serum and follicular fluid after hyperstimulation. Additionally, a significant positive correlation was found between posthyperstimulation serum and follicular fluid resveratrol levels. Follicular fluid resveratrol levels were also significantly lower in pregnant compared with non-pregnant women, but serum resveratrol levels were similar in both groups. These data show that SIRT1 and SIRT6 are significantly involved in human reproduction, and they may play a role in oocyte maturation and clinical pregnancy in vitro fertilization (IVF), but definitive conclusions cannot be drawn and studies should be continued. The results can be connected with their epigenetic damage by phosphorylation or by small interfering RNA, which may alter conformation of SIRT protein, binding to their substrates, catalytic activity, and immunoreaction [61,62].

Some authors also indicated the participation of SIRT1 in recurrent implantation failure (RIF) due to its known important role in cellular processes, including gene expression, cell cycle regulation, cell metabolism, oxidative stress response, apoptosis, cellular stress, inflammation, DNA repair, and aging [26,63]. RIF is an important problem during IVF—in intracytoplasmic sperm injection (ICSI) cycles. The maternal immune system as well as the mediators secreted from endometrium play a significant role in this interaction [64]. Engin-Ustun et al. [26] examined SIRT1 levels in women with RIF, healthy women who had conceived by IVF, and women with a 1-cycle failure of IVF, used as controls. They observed a low but statistically significant correlation between age and SIRT1 level. Additionally, in RIF patients, they revealed higher levels of this deacetylase than in non-pregnant women and healthy pregnant women, but the observed differences did not reach statistical significance. The authors suggest that the observed elevated SIRT1 levels may result from inflammation imbalance and oxidative stress in RIF patients [26]. In this light, the role of sirtuins, which can modulate various proteins and cellular routs, seems to be important.

Another sirtuin involved in female fertility appears to be SIRT3, the role and action of which were examined in follicular cells by Pacelle-Ince et al. [32]. In young women (aged below 35), an increased concentration of mRNA and SIRT3 concentration as well as its mitochondrial localization in granulosa cells (GCs) were observed, compared to cumulus oophorus cells in follicular cells taken from the same young women with normal ovarian reserve. However, the expression of SIRT3 mRNA and active protein was decreased in women below 35 with reduced ovarian reserve, as well as in women of an advanced maternal age (above 40) and reduced ovarian reserve, and it was confirmed both in granular cells and cumulus cells (CSs). The highest catalytic activity of this deacetylase was recorded in granular cells in healthy young women. It was lower in young women with a reduced ovarian reserve and the lowest in older women. However, this activity in the cumulus cells of young women did not differ between the healthy group and participants with disorders, but it was lower in older women. These alterations (in young women with reduced ovarian reserve or women of advanced age) are connected with reduced oocyte viability, possibly due to altered granulosa and cumulus cells metabolism, which was observed in these groups. These findings suggest that the mitochondrial SIRT3 protein may be implicated in these processes, as it is able to sense the metabolic state of the cell and alter mitochondrial protein function post-translationally [32].

**Figure 3:**
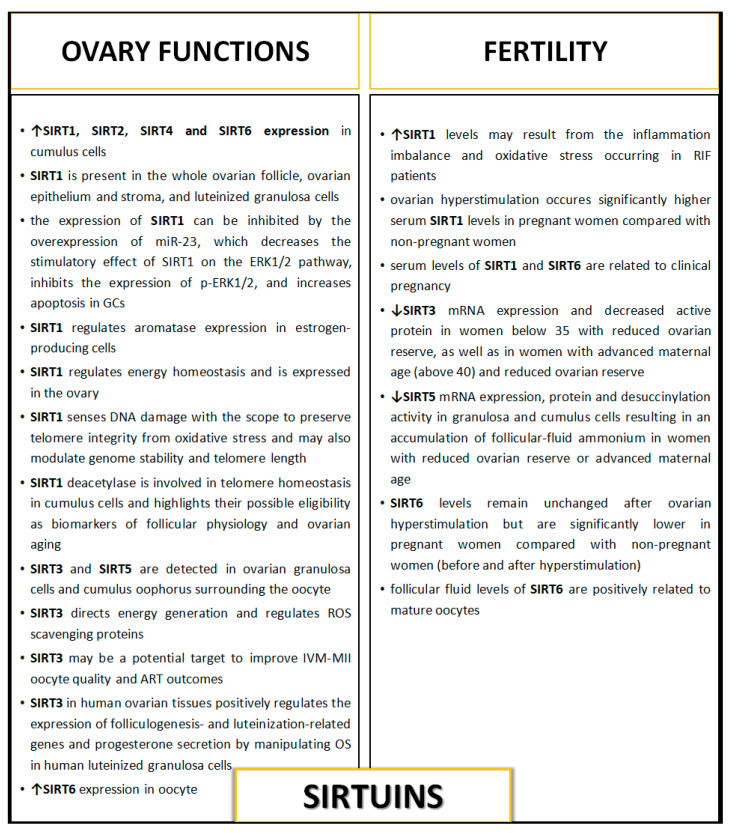
The influence of sirtuins on ovary function and female fertility. ART—assisted reproductive technology; DNA—deoxyribonucleic acid; GCs—granulosa cells; IVM-MII—in vitro matured metaphase II oocytes; mRNA—messenger RNA; miRNA—microRNA; OS—oxidative stress; RIF—recurrent implantation failure; ROS—reactive oxygen species.

The same research team [33] also drew attention to the role of SIRT5 in the modulation of ovarian reserves in women in terms of maternal age. The authors indicated that due to the fact that women with reduced ovarian reserve or advanced maternal age have an altered metabolic follicular microenvironment, and the fact that SIRT5 can sense cellular metabolic state and post-translationally alters protein function, its activity may directly impact oocyte viability and the outcome of pregnancy. They examined SIRT5 mRNA, protein, and protein activity in samples of surplus follicular fluid, granulosa, and cumulus cells derived from women of different maternal ages and ovarian reserves who were undergoing routine IVF treatment. Additionally, the presence of carbamoyl phosphate synthase 1 (CPS1), which is also a target of SIRT5, as well as follicular-fluid ammonium concentrations were measured. CPS1 is a ligase enzyme located in the mitochondria, which is also involved in the production of urea. Authors observed decreased SIRT5 mRNA, protein, and desuccinylation activity in granulosa and cumulus cells, which resulted in an accumulation of follicular-fluid ammonium in women with reduced ovarian reserve or advanced maternal age. They suggested that this can most likely be associated with alterations in CPS1 activity present in granulosa and cumulus cells, which is modulated by SIRT5 action—through the desuccinylation of CPS1, which modulates the levels of ammonium present in follicular fluid. This suggests an important role of SIRT5 in influencing oocyte quality and IVF outcomes [33]. The participation of sirtuins in female fertility is presented in Figure 1 and Figure 3.

### 4.1. Sirtuins in Ovary Function

It is known that the maintenance of ovarian reserve and the development of oocyte competence at all stages can be hampered by extrinsic and intrinsic factors, which may target the ovarian microenvironment during the entire reproductive lifespan [16,65]. Additionally, physiological levels of reactive oxygen species (ROS) have a pivotal role in the female reproductive system during folliculogenesis and oocyte maturation and may also affect the outcomes of assisted reproductive technology (ART) [30]. Sirtuins regulate important physiological events, including aging and cell metabolism, mainly by protecting cells and tissues from oxidative damage. Ovarian aging decreases the quality of oocytes through the induction of mitochondrial dysfunction and increases in DNA strand breaks by accumulation of ROS. However, the involvement of sirtuins in the regulation of oocyte quality with aging has yet not been determined, but their role in the modulation of appropriate proteins responsible for the proper action of oocyte and folliculogenesis is indicated [66,67,68].

In a study verifying the expression of seven sirtuin genes (*SIRT1-SIRT7*) in mouse ovaries, isolated oocytes, and cumulus cells, Okamoto et al. [69] found the expression of all sirtuins regulated in a cell-specific manner. Oocyte expressed high levels of SIRT6, whereas the expressions of SIRT1, SIRT2, SIRT4, and SIRT6 were high in cumulus cells. Comparing samples from young and aged mice, there was no difference between their oocyte levels of SIRT7 mRNA. However, SIRT2 and SIRT6 transcript levels were decreased in cumulus cells of aged mice. Authors suggested a possible association of SIRT2 and SIRT6 transcript levels in cumulus cells with impaired oocyte quality in aged mice, but they concluded that further studies dedicated to understanding the roles of these sirtuins in cumulus cells and oocytes could provide a better strategy to minimize aging-related decline in oocyte quality. This would be especially interesting in terms of the functioning of the female organism as well as female fertility and aging-associated pathologies, and is particularly important due to the potential clinical applications of sirtuin-activating compounds in older women, which could lead to novel approaches to the restoration of oocyte quality during aging [69].

Luo et al. [70] made an attempt to identify the microRNAs (miRNAs) in granulosa cells from the follicular fluid of patients with various levels of ovarian reserve function and the potential regulatory function of miR-23a in granulosa cell apoptosis also in terms of the role of SIRT1. The authors examined women undergoing IVF and intracytoplasmic sperm injection treatment. They identified 20 conserved and 3 novel miRNAs that were upregulated in the poor ovarian response (POR) group, and 30 conserved miRNAs and 1 novel miRNA that were upregulated in the polycystic ovary syndrome group. The results of bioinformatic analysis showed a complementary pairing between miR-23a and the 3′-untranslated region (UTR) of the SIRT1 mRNA, indicating that miR-23a can regulate SIRT1 protein expression at the posttranscriptional level in GCs. Overexpressing miR-23a can inhibit the expression of SIRT1, decrease the stimulatory effect of SIRT1 on the ERK1/2 pathway, inhibit the expression of p-ERK1/2, and increase apoptosis in GCs. Previous studies conducted by these authors confirmed that miR-23a targets SIRT1 and promotes apoptosis in GCs by inhibiting the ERK1/2 signaling pathway. This enables the new possibility of using miRNAs to regulate human GC apoptosis in vitro and indicate the important regulatory role of SIRT1 [70].

The involvement of sirtuins in the control of ovarian functions at various regulatory levels was also described by Sirotkin [1]. The author indicated that external and endocrine factors can affect female reproductive potential via the SIRTs-mammalian target of serine/threonine kinase, called the mammalian target of rapamycin (mTOR) system, mainly by its downregulation. In turn, via various hormones and pathways of growth factors, this can regulate basic ovarian functions (proliferation, apoptosis, secretory activity of ovarian cells, their response to upstream hormonal regulators, ovarian folliculo- and oogenesis, and fecundity). SIRT1 is present in the whole ovarian follicle, ovarian epithelium and stroma, and luteinized granulosa cells, while SIRT3 [32] and SIRT5 [33] have been detected in ovarian granulosa cells and cumulus oophorus surrounding the oocyte [32,33,71,72]. Sirotkin [1] indicated that the amount of SIRTs in ovarian cells is associated with their state and health. Sirtuins, SIRTs-related signaling molecules, and drugs regulating mTOR can be used in this manner for the characterization, prediction, and regulation of ovarian functions as well as for diagnostics and the treatment of ovarian disorders. SIRT1, SIRT3, SIRT5, and SIRT6 especially were indicated as potentially useful markers for the characterization and prediction of ovarian follicular development and related fecundity [1].

SIRT1 senses DNA damage with the scope to preserve telomere integrity from oxidative stress and may also modulate genome stability and telomere length. Additionally, cohesins SA1/SA2 mediate sister chromatid cohesion at telomere termini (SA1) and along chromatid arms (SA2) [73,74]. Valerio et al. [75] investigated SIRT1 and SA1/SA2 cohesin protein mRNA transcripts and DNA telomere sizing in cumulus cells to uncover their contribution to the physiology of the CCs. The authors [75] documented that a significant increase in SA1 and SA2 was disclosed in high responder women (>6 oocytes retrieved) compared to poor responders (<4 oocytes). Furthermore, significant positive correlations were observed between the transcript levels of these two cohesin molecules and, to a lesser extent, between telomere length and SA1 and SA2 mRNA levels. Moreover, SIRT1 expression was also significantly increased in high responders compared to poor responders. Significant correlations were found between SIRT1 and both cohesin molecules, and between SIRT1 and telomere length. However, in the group of women over 38 years of age, SIRT1 mRNA levels were twice as high as the levels recorded in the younger subject cohort. Valerio et al. [75] concluded that cohesins SA1/SA2 and SIRT1 deacetylase may be involved in telomere homeostasis in cumulus cells, and highlight their possible eligibility as biomarkers of follicular physiology and ovarian aging. However, the authors indicate the possible influence of ovarian stimulation protocol for IVF on obtained results as a limitation of the conducted study [75].

The first study investigating the role of the SIRT3 gene in mitochondrial biogenesis and the developmental competence of human in vitro matured oocytes was published by Zhao et al. [76]. Cytoplasmic immaturity in in vitro maturation (IVM) oocytes may lead to reduced developmental competence. Mitochondrial dysfunction results in the accumulation of free radicals and leads to DNA mutation, protein damage, telomere shortening, and apoptosis [77]. In turn, SIRT3 has emerged as a mitochondrial fidelity protein that directs energy generation and regulates ROS scavenging proteins. It regulates mitochondrial protein acetylation, which is capable of coordinating cellular responses to nutrient status and energy homeostasis [78]. Zhao et al. [76] conducted their study using in vivo matured metaphase II (IVO-MII) oocytes and IVM-MII oocytes donated by infertile women undergoing assisted reproductive technology cycles. A few oocytes, each in the germinal vesicle (GV), IVM, and IVO groups, were compared with respect to mRNA levels for SIRT1-7 mRNA, and five samples at each developmental stage were analyzed for SIRT3 mRNA. The authors observed that inefficient SIRT3 expression induced decreased mitochondrial DNA copy number and biogenesis, and therefore impaired the developmental competence of human in vitro maturation oocytes. In more detail, they showed a significant decrease in the abundance of SIRT mRNA and mitochondrial biogenesis in human IVM compared with IVO oocytes. The developmental potential of human IVM-MII oocytes to the blastocyst stage was also significantly reduced when SIRT3 mRNA was inhibited by siRNA, but it could be upregulated by the injection of SIRT3 mRNAs. Authors [76] concluded that the developmental potential of human IVM-MII oocytes is affected by decreased mitochondrial biogenesis and SIRT3 mRNA deficiency. These results collectively improve the understanding of important factors in oocyte maturation and highlight the utility of SIRT3 as a potential target to improve IVM-MII oocyte quality and ART outcomes. The results of their observations may help to improve the clinical application of the IVM procedure [76].

In the light of the known role of SIRT3 in OS regulation of ROS generation in oocytes [79], its participation in the regulation of the function and maturation of granulosa cells seems to be interesting. Fu et al. [80] examined SIRT3 expression and analyzed SIRT3-mediated oxidative response in human luteinized GCs. The authors observed that SIRT3 positively regulates the expression of folliculogenesis- and luteinization-related genes and progesterone secretion in human ovarian tissues by manipulating OS in human luteinized granulosa cells. The expression of SIRT3 in the GCs of the human ovary was observed. The mRNA levels of SIRT3, catalase, and superoxide dismutase 1 were upregulated by hydrogen peroxide in both human GCs and COV434 cell lines, and downregulated by human chorionic gonadotropin. A knockdown of SIRT3 markedly elevated ROS generation in human GCs. Additionally, SIRT3 depletion resulted in decreased mRNA expression of many agents connected with steroidogenesis (aromatase, 17β-hydroxysteroid dehydrogenase 1, steroidogenic acute regulatory protein, cholesterol side-chain cleavage enzyme, and 3β-hydroxysteroid dehydrogenase) in GCs and thus resulted in decreased progesterone secretion. Fu et al. [80] underlined the important clinical implications of folliculogenesis and luteinization processes in GCs—SIRT3, just by sensing and regulating the generation of ROS, and additionally through the activation of SIRT3 function, might help to sustain human reproduction by maintaining GCs as well as oocytes. The role of sirtuins in ovary functions is shown in Figure 1 and Figure 3.

### 4.2. Steroid Hormones and Sirtuins

The main role of steroid hormones such as progesterone, estradiol, and testosterone is to maintain normal reproductive function and body homeostasis [81]. Sirtuins are one of the many regulatory factors of steroid hormones, and take part in the process by signaling through a variety of molecular mechanisms, including acting as co-regulatory transcription factors, deacetylating histones in the promoters of genes with nuclear receptor-binding sites, directly deacetylating steroid hormone nuclear receptors, and regulating pathways that modify steroid hormone receptors through phosphorylation. Moreover, disruption of sirtuin activity may be a relevant step in the development of steroid hormone-refractory cancers [82].

Based on the latest research, it is indicated that proliferator-activated receptors/liver X receptor α (PPAR/LXRα) pathways are involved in the biosynthesis of steroid hormones. The regulation of steroidogenesis by PPAR/LXR is mainly connected with cholesterol efflux, which is stimulated by this signaling pathway [83]. However, the results of these studies are inconclusive, indicating that the regulatory effect of PPAR on steroid hormone synthesis may be dependent on the cell type, tissue, animal species, and its current functional state [84]. This is confirmed by studies concerning, for example, the influence of SIRT1 on PPARγ expression, where, depending on the type of adipose tissue, the expression increased—brown adipose tissue [85], or decreased—white adipose tissue [86]. In turn, SIRT2 inhibited adipogenesis by increasing the level of FOXO1 binding to PPARγ. This relationship between the SIRT2 and PPAR/LXRα pathways was verified by Xu et al. [81], who investigated the mechanism underlying SIRT2′s actions on the steroid hormone synthesis pathway in bovine GC cells. A bovine model is suitable as an animal model for the study of the physiological mechanism of human ovarian function. Bovine reproductive physiological characteristics are similar to those of humans (e.g., pregnancy cycle or ovarian structure). Additionally, SIRT2 is expressed in a wide range of bovine tissues. The authors found that SIRT2 plays critical roles in follicle development and maturation via maintaining steroid hormone homeostasis before mammalian pregnancy. It should also be highlighted that these results were the first to demonstrate that SIRT2 stimulated LXRα to regulate steroid hormone secretion in GCs. This finding suggests that the connection between SIRT2 and steroid hormone biosynthesis in GCs is more complex than in other cell types. The results demonstrated that the secretion of estradiol and testosterone is downregulated by treatment with the SIRT2 inhibitors Thiomyristoyl or SirReal2; however, this treatment also stimulated progesterone. Similar results were obtained with SIRT2 knockdown. Moreover, while SIRT2 knockdown might increase the stability of PPARα by acetylation, it does not increase the stability of PPARγ. In a study by Xu et al. [81], it was shown that SIRT2 knockdown might block PPARγ expression by mediating transcription factors (e.g., FOXO1, FOXO3a) or as-yet-unidentified actions in ovarian granular cells. This information may be useful for human reproductive health [81].

Some results of studies concerning the role of visfatin (Visf) as a cytokine hormone and enzyme—apart from its role in metabolic and immune disorders such as obesity and type 2 diabetes and in the regulation of numerous processes, including glucose and lipid metabolism, inflammation, and angiogenesis—also suggested its role in ovarian function [87,88,89]. Reverchon et al. [90] investigated molecular mechanisms in primary hGCs and in human ovarian granulosa-like tumor cell lines, involved in the regulation of visfatin expression in response to insulin sensitizers, metformin (MetF), and rosiglitazone, in human follicles and examined their potential connection with SIRT1 action. The authors showed that visfatin was expressed not only in hGCs and KGN cells but also in human cumulus cells and oocytes, and generally metformin and rosiglitazone increased visfatin mRNA (in a dose-dependent manner for MetF). Using the appropriate compounds (FK866, the visfatin inhibitor; compound C and aicar, inhibitor and activator of AMP-activated protein kinase (AMPK), respectively; sirtinol, an inhibitor of SIRT1), they showed that the MetF effects on visfatin expression were mediated through the AMPK/SIRT1 signaling pathways. The authors underlined that this was the first study to show that MetF activates SIRT1 activity in hGCs. SIRT1, like AMPK, regulates energy homeostasis and is expressed in the ovary. Moreover, a specific inhibitor of SIRT1, sirtinol, abolished MetF-induced visfatin mRNA, and protein expression, which suggests that not only AMPK but also SIRT1 is involved in this process. This creates a new important means of action for these agents in disturbances of human ovarian cells, including theca cells and oocytes, and probably also indicates its potential involvement in PCOS development [90].

## 5. Sirtuins in Gynecological Diseases

### 5.1. Sirtuins in Polycystic Ovary Syndrome

Polycystic ovary syndrome, a common and heterogeneous type of disorder, occurs in 5–10% of women of reproductive age. The main symptoms of PCOS are chronic anovulation with oligoamenorrhea, cystic follicles in the ovary, increased concentrations of luteinizing hormone (LH) as well as obesity, hyperandrogenism, insulin resistance (IR), and hyperinsulinemia, and finally infertility [91]. Additionally, oxidative stress, which participates in IR and hyperandrogenism with accompanying inflammation, is also indicated as an important element in PCOS development [10,92,93]. The participation of sirtuins, due to their proven role in many metabolic pathways, seems to be obvious and interesting in relation to such multifaceted disturbances.

A study conducted by Kiyak Caglayan et al. [29] among women with PCOS (aged 20–38 years) revealed that the mean SIRT1 level in the patients’ group was significantly higher (almost 1.5-times) than in the control group. There were no significant differences between the patients and control groups in fasting blood glucose, the insulin resistance index (HOMA-IR) or cholesterol, triglyceride, high-density lipoprotein (HDL), low-density lipoprotein (LDL), or C-reactive protein (CRP) levels. Moreover, the authors observed the presence of significant correlations between SIRT1 concentration and the formation of inflammation [29]. Rezaei et al. [39], based on the observed higher SIRT1 concentration in women with PCOS when compared to healthy subjects, examined the role of SIRT1 rs7895833 polymorphism in susceptibility to PCOS. The authors documented that in the dominant model for G allele (AG + GG vs. AA), AG + GG genotypes in SIRT1 rs7895833 gene polymorphism were strongly associated with increased risk of PCOS [39].

SIRT1 has also been reported to interact with BMAL1 (brain and muscle ARNT-like protein 1) and function in a circadian manner. Additionally, it was documented that SIRT1 regulates aromatase expression in estrogen-producing cells [1,94,95,96]. BMAL1 is necessary for fertilization and has been found to be essential to follicle growth and steroidogenesis. Zhang et al. [97] examined BMAL1 expression in their GCs by qRT-PCR in women diagnosed with PCOS and healthy individuals undergoing assisted reproduction. They observed significantly lower levels of BMAL1 expression in the PCOS group than in the group without PCOS. Analyzing estrogen synthesis and aromatase expression in KGN cell lines, the authors revealed that both were downregulated after BMAL1 and SIRT1 knockdown and, conversely, upregulated after overexpression treatments of these two genes in KGN cells. Additionally, they observed that both BMAL1 and SIRT1 had a mutually positive regulation, as did the phosphorylation of c-Jun NH_2_-terminal kinases (JNK). Furthermore, JNK overexpression increased estrogen synthesis activity and the expression levels of aromatase, BMAL1, and SIRT1. In KGN and hGCs, estrogen synthesis and aromatase expression were downregulated after treatment with JNK and SIRT1 inhibitors. In addition, BMAL1, SIRT1, and JNK expression levels were all downregulated. This suggests a BMAL1-SIRT1-JNK positive feedback cycle in this process, which points to the important role of BMAL1 in the development of PCOS. Additionally, this information may offer an instruction for clinical diagnosis, treatment, and prognosis for future patients with POCS and/or problems with fertilization [97].

### 5.2. Sirtuins in Endometriosis

Endometriosis is an estrogen-dependent pelvic inflammatory disease characterized by implantation and growth of endometrial tissue (glands and stroma) outside the uterine cavity. The etiology of endometriosis is still unclear: Sampson’s implantation theory [98,99], Mayer’s coelomic metaplasia theory from 1903, and the theory of induction [100] are three classic theories that have tried to designate the definitive pathogenetic mechanism of endometriosis, but they have failed to establish it. Additionally, the role of other factors in the development of endometriotic lesions, such as familiar tendency and genetic predisposition as well as oxidative stress and inflammation, was examined [101,102,103,104].

Endometriosis is also considered to be one of the causes of female fertility disturbances. It is documented that 40% of failed assisted reproductive technology cycles are due to undiagnosed endometriosis, and most women undertaking ART have never been tested for endometriosis before. The role of SIRT1 was previously mentioned. It is also noteworthy that women who over-express the oncogene BCL6 (B-cell lymphoma 6 protein) have a 47% reduction in live birth rate compared to women with normal BCL6 expression [105]. Yoo et al. [36,106] demonstrated phosphorylation of signal transduction and activators of transcription factor 3 (STAT3) in eutopic endometrium of infertile women with this disorder, leading to over-expression of the oncogene BCL6 and stabilization of hypoxia-induced factor 1 alpha (HIF-1α). The authors observed a correlation between SIRT1 and BCL6 expression in endometriosis. Additionally, they reported coordinated activation of KRAS (Kirsten rat sarcoma virus) and over-expression of SIRT1, a histone deacetylase and gene silencer, in the eutopic endometrium from women with endometriosis throughout the menstrual cycle. The authors showed that IL-6 activates JAK kinases and Ras-mediated signaling. Activation of KRAS, the key regulator of the Ras/ERK pathway, causes ectopic lesion establishment in the endometrium of mice. KRAS appears to be dysregulated in endometriosis [36,106]. Mice with conditional activation of KRAS in the progesterone-receptor-positive (PgR-positive) cells reveal an increase of SIRT1 expression in the endometrium compared to control mice. The expression of progesterone receptor target genes, including the Indian Hedgehog pathway genes, are significantly own-regulated in the mutant mice [36]. SIRT1 co-localizes with BCL6 in the nuclei of affected individuals; both proteins bind to and suppress the promoter of GLI1, a promoter target for both BCL6 and SIRT1, a critical mediator of progesterone action in the Indian Hedgehog pathway, by chromatinimmunoprecipitation analysis. In the eutopic endometrium, GLI1 expression is reduced in women with endometriosis. The authors suggest that KRAS, SIRT1, and BCL6 are coordinately over-expressed in the eutopic endometrium of women with endometriosis and likely participate in the pathogenesis of endometriosis [36].

The increased expression of SIRT1 in women with endometriosis and ovarian cancer associated with endometriosis was also demonstrated by Teasley et al. [37]. The observed increase in SIRT1 expression was related to the nuclear form and was correlated with the expression of KRAS in endometriosis-related ovarian cancer. These results suggest a role for KRAS and SIRT1 in endometriosis and endometriosis-related ovarian cancer. The authors conducted a study on human endometrial samples in groups of women with a regular cycle with endometriosis, ovarian cancer, and endometriosis-related ovarian cancer. The conclusions of their research indicate a significant role of KRAS and SIRT1 in the pathogenesis of endometriosis as well as ovarian cancer associated with endometriosis. The authors emphasize the special role of nuclear SIRT1 as a potential biomarker of endometriosis and ovarian cancer associated with endometriosis [37]. Following this lead that endometriosis is associated with abnormal cell proliferation and angiogenesis associated with increased estrogen secretion, some authors have suggested that endometriosis is a benign cancer-like disorder [38,107]. As already mentioned, sirtuins are one of the major regulators of several physiological processes, including metabolism and aging but also carcinogenesis. SIRT1, which is a crucial stimulator of cell growth and angiogenesis, is particularly involved in the latter process. In addition, sirtuins also regulate signaling pathways associated with steroid hormone receptors [108]. Taking into account the similarity of endometriosis to solid tumors and the related expression of SIRT1, Khazaei et al. [38] used noscapine, a natural alkaloid with anti-angiogenetic activity, in their study. It turned out that noscapine inhibited SIRT1 expression, cell proliferation and angiogenesis, and NO secretion in endometriotic explants. This proves that modulating SIRT1 expression may be beneficial in endometriosis, and, however, requires further research, nevertheless gives hope for finding a specific parameter for this non-specific disease, which could be a beneficial tool as a target modulator of clinical improvement in patients with endometriosis.

### 5.3. Sirtuins in Other Female Diseases

It is well documented that fetal malnutrition may predispose to type 2 diabetes. An alteration in gene expression profiles through epigenetic mechanisms is indicated as one of the forms taken by such disturbances [109]. Sirtuins, among their multiple functions, take part in the maintenance of glucose and lipid homeostasis and especially in the control of insulin secretion and sensitivity, the promotion of fat mobilization, influencing obesity-induced inflammation in macrophages, and the modulation of the activity of the circadian clock in metabolic tissues [110]. It has also been documented that mammalian sirtuins participate in the regulation of metabolic responses to nutritional input in multiple tissues and organs [111]. Rodgers et al. [112] have identified a molecular mechanism by which SIRT1 functions in glucose homeostasis as a modulator of PGC-1α (peroxisome proliferator-activated receptor gamma coactivator 1-alpha). This discovery has important implications for the fundamental pathways of energy homeostasis, diabetes, and lifespan. The authors confirmed the role of SIRT1 in controlling hepatic gluconeogenic/glycolytic pathways in response to fasting signals through the transcription co-activator PGC-1α. In fasting conditions, SIRT1 in the liver is induced by a pyruvate-mediated nutrient signaling response. Upon induction, SIRT1 interacts with PGC-1α and deacetylates at certain lysine residues in a NAD^+^-dependent manner. SIRT1 induces gluconeogenic genes and hepatic glucose production by PGC-1α but does not regulate the effects of PGC-1α on mitochondrial genes. In addition, SIRT1 modulates the effects of PGC-1α repression of glycolytic genes in response to fasting and pyruvate [112]. The same authors’ following work [113] demonstrated, in an in vivo experiment, that hepatic SIRT1 is a factor in systemic and hepatic glucose, lipid, and cholesterol homeostasis. After knockdown of SIRT1 in the liver, the authors observed mild hypoglycemia, increased systemic glucose and insulin sensitivity, and decreased glucose production. Decreased serum cholesterol and increased hepatic free fatty acid and cholesterol content were also demonstrated. These findings were closely correlated with decreased expression of gluconeogenic, fatty acid oxidation, and cholesterol degradation as well as efflux genes. In the next step of this experiment, the overexpression of SIRT1 reversed many of the changes caused by SIRT1 knockdown and was dependent on the presence of PGC-1α. However, the authors emphasize that most of the effects of SIRT1 were only apparent in the fasted state. These findings point out that hepatic SIRT1 is a significant factor in the regulation of glucose and lipid metabolism in response to nutrient deprivation. As the abovementioned pathways are dysregulated in metabolic diseases, SIRT1 may be a potential therapeutic target for the control of hyperglycemia and hypercholesterolemia [113]. Peeters et al. [114] suggested that polymorphism in SIRT1 increases the risk of obesity, and it was indicated that carriers of the variant C-allele of rs7069102 had a lower risk of obesity than non-carriers.

Based on data collected in a review by Kane and Sinclair [24], sirtuins appear to have a protective effect on obesity, although their effects are complex and may either be related or independent of insulin action and glucose regulation. This effect could be used for therapeutic and preventive purposes for diseases such as metabolic syndrome, insulin resistance, and type 2 diabetes, by increasing the activity of SIRT1 and SIRT6 or increasing the level of NAD^+^ [24]. As a nutrient-sensing histone deacetylase as well as through the deacetylation of histones, SIRT1 is involved in glucose and insulin metabolism, mainly by regulating the expression of various transcription factors. Thus, dietary factors influence the NAD^+^/NADH ratio, and regulating SIRT1 activity may influence the development of these disturbances in fetuses [115]. Botden et al. [116] investigated whether SIRT1 can influence fetal programming during malnutrition and analyzed the interaction between three SIRT1 single nucleotide polymorphisms and prenatal exposure to famine on type 2 diabetes risk in individuals of the Dutch Famine Birth Cohort. Authors reported that in the total population (exposed and unexposed), SIRT1 variants were not associated with type 2 diabetes. A significant interaction was found between two SIRT1 SNPs (rs7895833 and rs1467568) and exposure to famine in utero on the risk of type 2 diabetes. Minor alleles of these SNPs were associated with a lower prevalence of type 2 diabetes only in individuals who had been exposed to famine prenatally (for rs7895833 and for rs1467568). Authors concluded that SIRT1 may be an important genetic factor involved in fetal programming during malnutrition, influencing type 2 diabetes risk later in life and proving to be an interesting diagnostic or prognostic marker of the development of such diseases [116]. SIRT1 is known to be susceptible to intracellular fluctuations in the NAD^+^/NADH ratio and may influence type 2 diabetes risk through its known epigenetic effects and β-cell apoptosis [115,116].

Boyle et al. [35] investigated the association between SIRT3 activity and insulin resistance and systemic oxidative stress as prominent features of pregnancies complicated by maternal obesity or gestational diabetes mellitus (GDM). They measured mitochondrial enzyme activity and markers of oxidative stress (respiratory chain enzyme complexes I, II, III, and IV; citrate synthase (CS), aconitase, MnSOD and catalase, GSH:GSSG) in skeletal muscle tissue from pregnant women of normal weight (NW), obese pregnant women with normal glucose tolerance (NGT), and obese pregnant women with GDM, undergoing cesarean delivery (about 37 week gestation). The authors observed changes in levels of all examined parameters, especially in obese-NGT and obese-GDM women, which indicates increased oxidative stress. Additionally, mitochondrial SIRT3 mRNA content and enzyme activity were lower in the skeletal muscle of obese-NGT and obese-GDM women. Importantly, the acetylation of MnSOD, which is a SIRT3 target, was increased in obese-NGT and obese-GDM vs. NW women and was inversely correlated with SIRT3 activity, which indicates the existence of a mechanism for reduced MnSOD activity. Boyle et al. [35] concluded that skeletal muscle mitochondrial respiratory chain enzyme activity, decreased mitochondrial antioxidant defense, and reduced skeletal muscle SIRT3 activity observed in obese pregnant women may play a role in the increase of OS associated with pregnancies complicated by obesity and prove to be an area of preventive action among these patients.

## 6. Sirtuins in Typical Women’s Cancers

Typical female cancers have a poor overall survival rate in patients, and late disease presentation and chemo-resistance are the main factors that lead to the mortality of such patients [117]. Breast cancer is the second most common cause of female death after pulmonary cancer, and ovarian cancer has been described as the seventh most common female cancer [118,119]. Literature data have supported the important role of sirtuins in the functioning of the organism as well as in the proliferation, differentiation, cell cycle progression, and apoptosis of cancer cells [120,121,122].

### 6.1. Breast Cancer

In 2010, Yao et al. [44,45] reported that the inhibition of SIRT1 deacetylase suppresses estrogen receptor (ER) signaling, and additionally that the inhibition of estrogen signaling activates the NRF2 (nuclear factor erythroid 2-related factor 2, NF-E2-related factor 2) pathway in breast cancer. Scientific data demonstrate that estrogen is essential for mammary gland development as well as breast carcinogenesis. The biological functions of estrogen are elicited through estrogen receptor alpha (ERα)-mediated signaling pathways. It is known that ERα mediates estrogen-dependent gene transcription, which plays a critical role in mammary gland development, reproduction, and homeostasis [123]. It has been indicated that the inhibition of SIRT1 activity by sirtinol or SIRT1 knockout suppresses ERα expression by disrupting basal transcriptional complexes at the ERα promoter, which leads to the inhibition of estrogen-responsive gene expression [44]. Additionally, it was revealed that inhibition of ERα expression by the antiestrogen shikonin reverses the inhibitory effect of estrogen on NQO1 (NAD(P)H-quinone oxidoreductase 1) expression. The induction of NRF2-dependent detoxifying enzymes (e.g., NQO1) is considered an important mechanism of protection against estrogen-associated carcinogenesis. Estrogen recruits ERα and SIRT1 at the NQO1 promoter, leading to the inhibition of NQO1 transcription. NQO1 deficiency promotes estrogen-dependent tumor formation, and shikonin inhibits estrogen-dependent tumor growth in an NQO1-dependent manner in MCF-7 xenografts [45,46]. Yao et al. [45] and Santolla et al. [46] documented that SIRT1 is also involved in oncogenic signaling mediated by GPER (G-protein ER, formerly known as GPR30) in breast cancer by activating the EGFR/ERK/c-fos/AP-1 transduction pathway. Additionally, the authors reported that SIRT1 is involved in pro-survival effects elicited by E2 through GPER, such as the prevention of cell cycle arrest and cell death induced by the DNA-damaging agent etoposide. The aforementioned actions of estrogens were abolished by silencing GPER or SIRT1 as well as using the SIRT1 inhibitor—sirtinol [46].

The association between dysregulation of hormone metabolism and human breast cancer and sirtuin expression was described by Zhang et al. [47]. 17β-hydroxysteroid dehydrogenase type 4 (HSD17B4) catalyzes the conversion of estradiol (E2) to estrone (E1), and is associated with the pathogenesis and development of various cancers, especially those which are hormone-dependent. However, sirtuins participate in the regulation of various cell functions, and some authors [22,47,124] have shown that E1 upregulates HSD17B4 acetylation at lysine 669 (K669) and thereby promotes HSD17B4 degradation via chaperone-mediated autophagy (CMA), while a single mutation at K669 reverses the degradation and confers migratory and invasive properties to MCF7 cells upon E1 treatment. CREBBP (CREB-binding protein) dynamically controls the K669 acetylation level of HSD17B4 in response to E1. CREBBP overexpression distinctively increased the K669 acetylation level and inversely decreased the level of HSD17B4 protein. More importantly, K669 acetylation is inversely correlated with HSD17B4 in human breast cancer tissues [47]. In terms of sirtuins, the overexpression of SIRT3, but not other SIRTs, decreased the K669 acetylation level of endogenous HSD17B4 [47]. HA-tagged SIRT3 consistently and significantly decreased K669 acetylation levels of exogenous and endogenous HSD17B4 in HEK293T cells, whereas the inactive H248Y mutant of SIRT3 had no effect on HSD17B4 acetylation. These results demonstrate that SIRT3 is responsible for K669 acetylation of HSD17B4. The authors concluded that K669 acetylation of HSD17B4 promotes its degradation and observed that SIRT3 overexpression increased HSD17B4 protein. SIRT3 knockdown consistently reduced HSD17B4 protein, which was blocked by NH_4_Cl treatment [47]. Zhang et al. [47] concluded that their study was the first to reveal a crosstalk between acetylation and CMA degradation in HSD17B4 regulation, the role of SIRT3, and the critical role of this regulation in the malignant progression of breast cancer.

Decreased mitochondrial SIRT3 expression is a potential molecular biomarker associated with poor outcomes in breast cancer. Desouki et al. [48] analyzed SIRT3 expression in human breast cancer tissue and examined the relationship between SIRT3 expression and outcome in patients with breast cancer. They revealed that SIRT3 expression was significantly lower in neoplastic, compared with normal, breast epithelium, including normal epithelium adjacent to tumors. Patients with breast cancer in the lowest quartile values of SIRT3 expression had a significantly shorter loco-regional relapse-free survival. Additionally, low SIRT3 expression was associated with reduced loco-regional relapse-free survival in all breast cancer subtypes analyzed, including ER+, ER-, HER2+, and basal subtypes. The authors underlined the importance of SIRT3 as a tumor suppressor protein in breast cancer and suggest that SIRT3 may be a potential molecular biomarker for the identification of high-risk patients across all molecular subtypes of breast cancer [48].

Reactive oxygen species levels consequential to the functional alteration of key mitochondrial attributes contribute to carcinogenesis, either directly via oxidative DNA damage infliction or indirectly via the activation of oncogenic signaling cascades [49]. Earlier Kumari Kanchan et al. [125] reported that the activation of a key oncogenic signaling cascade via the mammalian target of rapamycin (mTOR) signaling complex-2 (mTORC2) owing to estrogen receptor (ER-α)-dependent augmentation of O_2_^•−^ within the mitochondria of 17-β-estradiol (E2)-stimulated breast cancer cells. Later, Lone et al. [49] observed ER-dependent transient inhibition of manganese superoxide dismutase (MnSOD) catalytic function in breast cancer cells and that catalytic function of MnSOD is tightly regulated at the post-translational level. MnSOD is the principal mitochondrial attribute governing mitochondrial O_2_^•−^ homeostasis, which raises the possibility that its functional alteration could be instrumental in augmenting mitochondrial O_2_^•−^ levels in breast cancer cells. In the aspect of post-translational modifications, such as phosphorylation, nitration, and acetylation, which represent key regulatory means governing the catalytic function of MnSOD, the authors revealed that acetylation at lysine-68 (K68) inhibits MnSOD catalytic activity and thus represents an important post-translational regulatory mechanism in human cells. Moreover, they demonstrated the occurrence of a direct physical interaction between ER-α and MnSOD in human breast cancer cells, which in turn was associated with potentiated acetylation of MnSOD at K68. The authors also observed diminished interaction of MnSOD with SIRT3, the key mitochondrial deacetylase that deacetylates MnSOD at critical K68 and thereby activates it for scavenging O_2_^•−^. Consequently, compromised deacetylation of MnSOD at K68 leading to its inhibition and a resultant buildup of O_2_^•−^ within the mitochondria culminated in the activation of the mTORC2 signaling pathway. The authors documented that human breast cancer tissue specimens exhibited a positive correlation between acetyl-MnSODK68 levels and phospho-Ser2481 mTOR levels. Lone et al. [49] concluded that their results provided a mechanistic link for ER-α-dependent O_2_^•−^ potentiation and resultant mTORC2 activation in breast cancer cells. Additionally, the authors underlined their exposition of the crosstalk of ER-α with MnSOD post-translational regulatory mechanisms. Moreover, they also unraveled the regulatory role of ER/MnSOD interaction as an important control switch for redox regulation of ER-α-responsive oncogenic signaling cascades [49].

### 6.2. Endometrial Cancer

Some studies have suggested that SIRT1 may promote endometrial tumor growth [50,51], and others described its potential role in endometriosis and embryo endometrial receptivity [12,126]. A large study conducted by Bartosch et al. [53] examined the mRNA expression of sirtuins (SIRT1–7) in various types (Type I, Type II, and one mixed EC) of endometrial cancers (EC) and non-neoplastic endometria (NNE) by quantitative real-time PCR, and their protein expression was evaluated by immunohistochemical analysis using the Allred score, which provided interesting information. Authors documented that, compared to NNE, EC showed statistically significant SIRT7 mRNA overexpression, whereas SIRT1, SIRT2, SIRT4, and SIRT5 were significantly underexpressed. However, no significant differences were observed for SIRT3 and SIRT6. Additionally, Type II EC displayed lower SIRT1 and SIRT3 transcript levels than Type I EC. Considering protein expression, the SIRT1 immunostaining median score was higher in EC compared to NNE epithelium, while SIRT7 expression was lower in EC. The authors found no significant correlations between SIRT1/7 immunoexpression and histological subtype, grade, lymphovascular invasion, or stage. Bartosch et al. [53] underlined that the obtained results show that sirtuins are deregulated in EC, and that this observed diversity of expression patterns suggests that sirtuins may have distinctive roles in endometrial cancer, similarly to what has been described for other cancer models.

Asaka et al. [51] paid attention to the role of SIRT1 in endometrial carcinoma, taking into account the fact that a high-calorie diet is a well-known risk factor for endometrial carcinoma and SIRT1 is induced by caloric restriction, and regulates various cellular functions such as DNA repair, cell survival, metabolism. In the context of the association of a high-calorie diet with endometrial carcinoma, the authors supposed that SIRT1 might be downregulated in the normal endometrial glandular cells of obese women. Surprisingly, they did not observe a correlation between the expression of SIRT1 and body mass index (BMI). In contrast, the immunohistochemical expression of SIRT1 was significantly higher in endometrial carcinoma than in normal endometria cases regardless of BMI, and its overexpression was associated with shorter survival among patients. In vivo experiments conducted by Asaka et al. [51] showed that SIRT1 accelerated the proliferation of different endometrial carcinoma cell lines (HHUA, HEC151, and HEC1B). Additionally, the observed SIRT1 overexpression significantly enhanced the resistance to cisplatin and paclitaxel in HHUA cells. The authors documented that although p53 is an important target protein for SIRT1 action, the selective inhibitor of this deacetylase—EX527 significantly suppressed the proliferation and cisplatin resistance of three endometrial carcinoma cell lines regardless of the p53 mutation status. Moreover, SIRT1 overexpression in HHUA cells accelerated tumor growth and cisplatin resistance in nude mice, and EX527 significantly suppressed the growth of tumors of HHUA and HEC1B cells. The adverse effect of EX527 was not observed in these mice. The authors concluded that SIRT1 is involved in the acquisition of the aggressive behavior associated with endometrial carcinoma, and it can be a promising therapeutic target candidate, underlying that the EX527, as a SIRT1 inhibitor, may be a useful agent for the treatment of endometrial carcinoma [51].

SIRT1 plays a dual role as a tumor promoter as well as a tumor suppressor. Its involvement in tumorigenesis may be due to its diverse distribution in various tissues and different upstream and downstream regulatory factors that regulate its function [127,128]. Sterol regulatory element-binding protein 1 (SREBP1) belongs to the family of the basic helix-loop-helix leucine zipper family of DNA-binding transcription factors, and it has been indicated that SIRT1 acts on it. SREBP1 can regulate most enzymes involved in fatty acid biosynthesis, such as acetyl-CoA carboxylase, fatty acid synthase, Elovl-6, and stearoyl-CoA desaturase [129]. However, it is indicated that obesity is an established epidemiological risk factor in EC, lipogenesis is increased in cancer cells, and the expression of SREBP1 has been observed to be elevated in various types of cancer [130,131,132]. Lin et al. [50] revealed significantly higher expression levels of SIRT1 in endometrial cancer and observed the correlation between SIRT1 expression and SREBP1 in EC, which indicated that SIRT1 could stimulate endometrial tumor growth through the lipogenic pathway. This study was conducted in cases of fresh uterine endometrial adenocarcinoma and involved the examination of adjacent normal endometrial tissues obtained from patients who had undergone initial hysterectomy, as well as in human EC cell lines (Ishikawa, ECC, RL95-2, KLE). The authors also revealed that knockdown of SIRT1 could downregulate the expression of SREBP1 and suppress cell proliferation. This demonstrated that SIRT1 may play a role as a tumor promoter in EC and can promote endometrial tumor growth by promoting lipogenesis, which is why SIRT1 may be regarded as the target of the management of EC [50].

Recently Mao et al. [55] examined SIRT7 expression in tissue samples derived from 5 female endometrial cancer patients as well as in endometrial cancer cell lines and cultures (KLE, RL95-2, AN3CA, and Ishikawa), and made an interesting observation that SIRT7 knockdown markedly inhibits the growth of endometrial cancer cells by inducing apoptosis via the NF-κB (nuclear factor kappa B) signaling pathway. SIRT7 was overexpressed in endometrial cancer cells when compared with normal endometrial cells, and, importantly, its downregulation inhibited the growth and invasiveness of endometrial cancer cells. Additionally, the authors observed that the knockdown of SIRT7 causes an increase in the sensitivity of endometrial cancer cells to cisplatin treatment in vitro. This clearly indicates SIRT7 participation in endometrial cancer formation and its susceptibility to treatment. The authors indicated the nuclear factor (NF)-κB signaling pathway as the molecular mechanism of this action. The knockdown of SIRT7 inhibited NF-κB expression and resulted in decreasing expression of NF-κB target anti-apoptotic proteins: Bcl-xl, Bcl-2, and Mcl-1. SIRT7 knockdown also resulted in an increase of pro-apoptotic NF-κB target proteins Caspase-3, Bad, and Bax. Mao et al. [55] underlined the important role of SIRT7 in endometrial cancer, and suggested that SIRT7 may be a potential therapeutic target for endometrial cancer therapy.

On the other hand, Tekin et al. [52] observed a lack of association between other sirtuin—SIRT1—gene variants and endometrial cancer. Using paraffin-embedded endometrium specimens from endometrial cancer patients and healthy control subjects, the authors examined the correlation between rs7895833, rs7069102, and rs2273773 polymorphisms of SIRT1 gene and endometrial cancer. On the basis of the PCR-CCTP method and single-nucleotide polymorphism (SNP) analysis, they revealed no significant correlation with endometrial cancer. However, due to the relatively small number of subjects in the examined group, indicated as a limitation of their study, the authors showed that their results did not reflect the exact contribution of polymorphism in the development of disease. They concluded that this aspect is very interesting; however, further studies need to be performed with a higher population number, which will allow confirmation whether polymorphism is a potential novel genetic marker of endometrial cancer [52].

Some studies indicated that SIRT4, apart from regulation of glutamine metabolism, also serves as a tumor suppressor. Chen et al. [54] reported that decreased SIRT4 concentration, observed in tissues derived from endometrioid adenocarcinoma patients, are associated with advanced stages on the AJCC scale. AJCC is a classification staging system developed by the American Joint Committee on Cancer for describing the extent of disease progression in cancer patients [133]. The authors observed significantly lower SIRT4 concentration in endometrioid adenocarcinoma than in non-neoplastic tissue counterpart. Moreover, lower SIRT4 expression levels were observed in advanced AJCC stages of development, and they were significantly different in comparison to those observed in earlier disease stages [54].

### 6.3. Ovarian Cancer

Some authors indicated that SIRT1 is overexpressed in women with ovarian carcinoma [13,41]. Mvunta et al. [13] observed that the expression of SIRT1 was higher in endometrioid, mucinous, and clear-cell carcinomas than in cysts of normal ovaries, but not in serous carcinoma. However, the expression of SIRT1 in carcinoma ovarian patients did not correlate with age, stage, location of metastasis, or capsular penetration. The authors concluded that elevated SIRT1 expression was a significant predictor of shorter survival of ill patients (confirmed by univariate as well as multivariate survival analyses), regardless of the tumor stage. The results of this study suggest a positive influence of SIRT1 on the reduction of OvCa development and its potential as a novel therapeutic target [13].

The association between SIRT1, ovarian cancer, and hypoxia was checked by Qin et al. [40]. Hypoxia is a common characteristic of many malignant tumors, and increased expression of hypoxia-inducible factor 1-alpha (HIF-1α) predicts the poor prognosis of ovarian cancer. However, SIRT1 was found to be the downstream target gene of HIF-1α. The authors used cancer stem cell-like (CSC) properties, a small subpopulation of cancer cells, in human ovarian cancer cell lines SKOV3 and HO8910 and indicated that CSCs have been associated with resistance to chemo- and radio-therapy in cancer treatment. Qin et al. [40] reported the correlation between hypoxia and cancer stem cell-like properties in human ovarian cancer cell lines SKOV3 and HO8910. They found that as a downstream target gene of HIF-1α, SIRT1 was involved in the promotion of cancer stem cell-like features in ovarian cancer cells by hypoxia. SIRT1 was overexpressed in ovarian cancer cell exposure to hypoxia condition, and the NF-κB signaling pathway was involved in hypoxia-induced SIRT1 up-regulation—HIF-1α promoted CSCs-like features by increasing SIRT1 expression via NF-κB signaling pathway activation. SKOV3 cells transfected with SIRT1 siRNA exhibited a significant decrease in chemo-resistance and EMT (epithelial to mesenchymal transition) phenotype. These results indicated that SIRT1 was involved in the increase of CSC-like features in ovarian cancer cells induced by HIF-1α. As a final conclusion, the authors summarized that HIF1α and SIRT1 might serve as potential therapeutic targets for ovarian cancer [40], establishing an interesting connection between different pathways of observed disturbances and cancer pathogenesis.

Recent studies on human ovarian cancer cells (SKOV3) conducted by Hou et al. [42] concerned the significance of SIRT3 in the poor prognosis of ovarian cancer, which is mainly caused by chemotherapy resistance. It was documented that the Bcl-2 inhibitor ABT737 can significantly improve the effect of cisplatin and induce mitochondrial pathway apoptosis. However, the mechanism of ABT737 increasing sensitivity to cisplatin in ovarian cancer cells remains unclear. As a mitochondrial histone deacetylase, SIRT3 is involved in the regulation of mitochondrial function in cancers. The authors reported that cisplatin, accompanied with ABT737, promoted apoptosis and decreased the mitochondrial membrane potential of ovarian cancer cells. They indicated that ABT737 enhanced the sensitivity of ovarian cancer cells to cisplatin, which was partially achieved by activating SIRT3 to regulate the mitochondrial fission process. This establishes SIRT3 as a potential therapeutic target for ovarian cancer [42].

Another possible novel therapeutic target for ovarian cancer therapy is SIRT6. Zhang et al. [43] examined the mRNA and levels of SIRT6 in human ovarian cancer tissues and normal tissues as well as in cell lines. The authors reported that SIRT6 expression (at the mRNA and protein levels) was significantly reduced in human ovarian cancer tissues compared to normal tissues. Furthermore, they showed that overexpression of SIRT6 inhibited the proliferation of ovarian cancer cells SKOV3 and OVCAR3. By contrast, down-regulation of SIRT6 enhanced ovarian cancer cell growth. In addition, they revealed that SIRT6 suppressed the expression of Notch 3 through downregulation of its expression both at the mRNA and protein levels in ovarian cancer cells [43]. Notch 3 is proposed as a candidate oncogene, the Notch 3 signaling pathway as involved in the tumor progression of ovarian carcinoma, and higher Notch 3 expression as a possible independent, poor prognostic factor [134,135].

### 6.4. Uterine Cancer

Uterine sarcomas are uncommon and aggressive tumors comprising 3–7% of all uterine malignancies. Stage and histology have the strongest bearing on survival; leiomyosarcoma has the worst survival, whereas adenosarcoma has the best prognosis [136]. It is indicated that miRNA may be associated with the risk of uterine sarcoma. Tong et al. [56] examined the levels of serum miR-152, miR-205, miR-222, miR-24, miR-150, and SIRT1 in patients with uterine sarcoma and healthy subjects by quantitative real-time polymerase chain reaction (qRT-PCR). Additionally, HeLa cells were transfected with the mimics of miR-152 and miR-24, and the autophagy rates, the levels of SIRT1, as well as acetylation of microtubule-associated protein 1A/1B-light chain 3 (LC3) were measured. The authors documented that the levels of miR-152, miR-24, and SIRT1 significantly decreased, while the levels of miR-205, miR-222, and miR-150 were significantly higher in patients when compared to the healthy subjects. Additionally, Kaplan–Meier analysis demonstrated that uterine sarcoma patients have better survival rates with high-level miR-152 and miR-24 (with a five-year overall survival rate of 21.8% and 67.5%, respectively). Mimics of miR-152 and miR-24 induced autophagy by increasing the level of SIRT1, which deacetylated LC3. Tong et al. [56] concluded that observed altered miRNA species in uterine sarcoma are linked to disease stage. Moreover, a new molecular mechanism of disease development, by which miR-152 and miR-24 would promote autophagy by activating SIRT1 and deacetylating LC3, was indicated [56].

## 7. Resveratrol—A Promising Cure for Female Diseases

Recently, researchers have been interested in the role and usefulness of resveratrol in the normalization of disorders related to aging of the ovaries or impairment of their function. RSV, as a natural compound, is known from its protective effects against aging. It has also been reported that RSV has similar positive effects on the human metabolism as caloric restriction [137]. Banaszewska et al. [138] documented that in women with PCOS resveratrol, applied over a period of 3 months, significantly reduced ovarian and adrenal androgens. RSV reduces androgen concentration (e.g., testosterone) produced at too high amounts by ovaries and the adrenals by more than 20%, and it decreases fasting insulin levels by 32% [138]. Marti et al. [139] in their studies examined the mechanism of RSV action on steroidogenesis and ovarian function using the adrenocortical H295R human cell line and documented that RSV is able to inhibit protein expression as well as enzyme activities of cytochrome CYP17 and CYP21, but it did not alter CYP17 and CYP21 mRNA expression or protein degradation. They also found that only SIRT3 mRNA expression is altered by RSV, but SIRT1, 3 and 5 overexpression did not result in a change in the steroid profile of H295R cells, which suggests that resveratrol may not significantly engage sirtuins to modulate steroid production. Marti et al. [139] concluded that their cell line study makes resveratrol a candidate for the treatment of hyperandrogenic disorders such as PCOS.

Taking into account that endometriosis is known as a chronic inflammatory disease, together with SIRT1′s known role in regulation of inflammation and the expression of inflammatory cytokines, Taguchi et al. [12] studied the anti-inflammatory effects of SIRT1 on endometriosis development and the action of resveratrol or sirtinol (potent activator and inhibitor of SIRT1, respectively). SIRT1 was expressed in endometriotic stromal cells (ESC) and normal endometrial stromal cells (NEC) and resveratrol suppressed TNF-α-induced IL-8 released from the ESC in a dose-dependent manner, while sirtinol increased IL-8 release. The authors concluded that these observed opposing effects of SIRT1-related agents suggest that IL-8 release from ESC is modulated through the SIRT1 pathway, and resveratrol anti-inflammatory effects are more prominent in ESC than in NES, which suggests its potential relevance in the treatment of endometriosis [12]. Recently, Dull et al. [140] emphasized the therapeutic effects of resveratrol on endometriosis via anti-inflammatory and anti-angiogenic pathways, especially in the light of current theories that propose that chronic inflammation can influence the development of endometriosis. The authors also highlighted the significance of dietary phytochemicals and their effect on various inflammatory diseases, and concluded that nowadays more and more scientific and clinical studies are focused on the analysis of nutraceuticals, such as phenolic compound resveratrol, which can be taken into consideration as a new innovative drug in the prevention and treatment of this disease [140].

Shirane et al. [126] indicated that regulation of SIRT1 can determine the initial step of endometrial/uterine receptivity by controlling E-cadherin expression on endometrial carcinoma cell lines. They showed that exogenous expression of SIRT1 significantly enhanced E-cadherin expression, while siRNA-mediated depletion of endogenous SIRT1 resulted in a significant reduction of E-cadherin expression. The application of an SIRT1 activator, resveratrol, elevated E-cadherin expression in a dose-dependent manner, while SIRT1 repressors (nicotinamide and sirtinol) exhibited a dose-dependent reduction of E-cadherin expression. Additionally, the authors indicated that both forced expression and activation of SIRT1 promote E-cadherin-driven reporter gene constructs, and SIRT1 is localized at the E-cadherin promoter containing E-box elements in endometrial carcinoma cell lines. Using an in vitro model of embryo implantation, Shirane et al. [126] demonstrated that exogenous SIRT1 expression and stimulation of SIRT1 activity resulted in the endometrial carcinoma cell line becoming receptive to JAR cell spheroid attachment. Furthermore, resveratrol enhanced E-cadherin and protein glycodelin expression at sites of intercellular contact, suggesting an additive role of resveratrol in promoting implantation and its future application as a protective agent [126].

Pacelle-Ince et al. [32] examined the degree of acetylation of glutamate dehydrogenase (GDH), which is a mitochondrial enzyme that participates in the urea cycle (it converts glutamate to α-ketoglutarate and vice versa). An increase in the degree of GDH acetylation in granular cells was observed in young sick women compared to healthy women, and this increase was even higher in older women. There were no differences in the group of younger women in the cumulus cells, but in the older group, the degree of acetylation of this enzyme was higher. The differences in the degree of GDH acetylation were due to differences in the activity of SIRT3 in these cells, because this enzyme is a SIRT3 substrate. The action of this deacetylase on glutamate GDH has been confirmed in granulosa cells obtained from young healthy women by subjecting them to resveratrol and nicotinamide action (as known sirtuins activator and inhibitor, respectively). In the presence of resveratrol, GDH activity increased and decreased after the inhibitor application. The above observations confirm that the catalytic activity of SIRT3 is reduced in women with reproductive problems, which may affect the transformation of many mitochondrial proteins in cells that are substrates for this sirtuin. These disorders, e.g., GDH activity, may have a negative effect on cell function—in this case, oocyte viability [32]. Authors concluded that perturbations caused by SIRT3 via post-translational protein modification in human granulosa and cumulus cells may be a causative factor in the decline of oocyte viability in women with reduced ovarian reserve and advanced maternal age. It is important because follicular metabolism is essential for the development of a competent oocyte, and therefore alterations in SIRT3 as well as its targets in granulosa and cumulus cells may alter the follicular environment and impact oocyte health. Knowledge about such perturbations may lead to novel therapies for improving mitochondrial metabolism that would specifically target the deacetylation of GDH in the oocyte and follicular cells of women undergoing IVF treatment [32].

## 8. Conclusions

Concluding, despite many studies, sirtuins are still an interesting research target, also in the context of women’s gynecological health. Sirtuins play a significant role in both the formation and the course of many gynecological diseases. Their role is particularly important and well documented in the course of cancer developing within the female reproductive organs; however, they are also widely investigated in terms of disturbances observed in the ovary and oocyte as well as in follicular fluid. Moreover, sirtuins participate in some gynecological disturbances as regulative factors in pathways associated with insulin resistance, glucose and lipid metabolism disorders.

## Figures and Tables

**Table 1:** Sirtuins in gynecological diseases.

Disease	Type of Analyzed Sirtuin	Reference
Fertility problems	SIRT1 SIRT3 SIRT5 SIRT1, SIRT6	Engin-Ustun et al. [26] Pacella-Ince et al. [32] Pacella-Ince et al. [33] Bódis et al. [34]
Gestational diabetes mellitus	SIRT3	Boyle et al. [35]
Endometriosis	SIRT1 SIRT1 SIRT1 SIRT1	Taguchi et al. [12] Yoo et al. [36] Teasley et al. [37] Khazaei et al. [38]
Polycystic Ovary Syndrome	SIRT1 SIRT1	Kiyak Caglayan et al. [29] Rezaei et al. [39]
Ovarian cancer	SIRT1 SIRT1 SIRT1 SIRT3 SIRT6	Mvunta et al. [13] Qin et al. [40] Jang et al. [41] Hou et al. [42] Zhang et al. [43]
Breast cancer	SIRT1 SIRT1 SIRT3 SIRT3 SIRT3	Yao et al. [44,45] Santolla et al. [46] Zhang et al. [47] Desouki et al. [48] Lone et al. [49]
Endometrial cancer	SIRT1 SIRT1 SIRT1 SIRT1-7 SIRT4 SIRT7	Lin et al. [50] Asaka et al. [51] Tekin et al. [52] Bartosch et al. [53] Chen et al. [54] Mao et al. [55]
Uterine cancer	SIRT1	Tong et al. [56]

## Data Availability

Not applicable.
